# Prescription Department

**Published:** 1891-12

**Authors:** 


BRONCHITIS.—

R Acidi salicylici, dr. ij.
Ammoni carbonat, dr. vj.
Syrupi simplicis, oz. iij.
Aquae, ad oz. viij. M.

Sig. A dessertspoonful every hour or two to an adult.—Fliesberg, Annual Univ. Med. Sci.

R Vini ippecac. dr. ij.
Liq. potas eitratis, dr. iv.
Tinct. opi camph.
Syrup acaciae, aa oz. j. M.

Sig. A tablespoonful three times daily. (In first stage of ordinary acute bronchitis.)—DaCosta.

R Acidi hydrocyanici dil. m xvj.
Syr. pruni virginian.
Aq. camphorae, aa oz. j. M.

Sig. A teaspoonful every two or three hours. (In violent, troublesome cough.)—Hartshorne.

R Tinct. sanguinarae, dr. j.
Tinct. lobeliae, dr. j.
Vini ipecac, dr. ij.
Syr. tolutan, oz. ss. M.

Sig. A teaspoonful every 3 hours. Bartholow.

PLEURISY—

R Potassi acetatis, gr. xv.
Spt. aetheris nitrosi, dr. ss.
Vini ipecacuhana, gtt. iij.
Syr. tolutani, dr. ss. M.

Sig. To be taken four times daily. (In subacute pleurisy.)—DaCosta.

R Tinct, opii deodoratae, gtt. xx.
Tinct. digitalis, gtt. xvj.
Syr. pruni virginiae, oz. j.
Aquae, oz. iss. M.

Sig. A teaspoonful every 3 hours for a child eighteen years old. (In first stage.)—J. Lewis Smith.

PNEUMONIA—

R Quinae sulphatis, dr. ss.
Acidi sulphurici aromat., dr. iss.
Olei caryophylli, gtt. iv.
Mucil acaciae, oz. j.
Aquae menthae pip, ad oz. iv.

M. Sig A teaspoonful or two every three or four hours. (In asthenic pneumonia.)—Hartshorne.

Pneumonia—

R Ammonii muriatis, dr. j.
Extract glycyrrhizae, dr. j.
Spts. aetheris sulph., dr. ij.
Aquae, oz. iv. M.

Sig. A tablespoonful every two or three hours. (In advanced stages of pneumonia.)—Waring.

R Tinct. veratri viridis, m. xl.
Spts. aetheris nitrosi, dr. vj.
Liq. potassii vitratis, dr. ivss.
Syr. zingiberis, ad oz. vj. M.

Sig. A tablespoonful every 3 hours. (In the early stage.)—DaCosta.

R Potassii iodidi, dr. j.
Ammonii muriatis, dr. iss.
Mist. glycyrrhizae co., oz. vj.

M. Sig. A tablespoonful four times daily, to promote absorption, together with blisters to the chest.—DaCosta.

Constipation—

R Resinae podophylli, gr. ij.
Quinae sulphat.,
Ext. aloe socot, aa gr. viij.
Fellis bovini, gr. xvj. M.

In pil. no. xvi div.

Sig. One or two at bed-time.—Goodell.

Convulsions—

R Chloral hydrat, gr. xv–xxx.
Syr. acaciae, oz. j.
Aquae, ad oz. iv. M.

Sig. Inject a tablespoonful into the rectum, and repeat in fifteen or twenty minutes if required. (Half the quantity if by mouth.—Widerhofer.

Eczema and Psoriasis—

To remove the abnormal scaly formation of the skin and assist other treatment, the following has been found remarkably successful:

R Papoid
Boracic acid, aa dr. ss.
Glycerine.
Aqua camphor, aa q. s. to make a paste.

Apply night and morning, allownig the paste to dry on the skin.

